# Safety of Semaglutide

**DOI:** 10.3389/fendo.2021.645563

**Published:** 2021-07-07

**Authors:** Mark M. Smits, Daniël H. Van Raalte

**Affiliations:** Diabetes Center, Department of Internal Medicine, Amsterdam University Medical Center, Amsterdam, Netherlands

**Keywords:** glucagon-like peptide-1 receptor agonist (GLP-1RA), oral, subcutaneous, semaglutide, type 2 diabetes, safety

## Abstract

The glucagon-like peptide-1 receptor agonist (GLP-1RA) semaglutide is the most recently approved agent of this drug class, and the only GLP-1RA currently available as both subcutaneous and oral formulation. While GLP-1RAs effectively improve glycemic control and cause weight loss, potential safety concerns have arisen over the years. For semaglutide, such concerns have been addressed in the extensive phase 3 registration trials including cardiovascular outcome trials for both subcutaneous (SUSTAIN: Semaglutide Unabated Sustainability in Treatment of Type 2 Diabetes) and oral (PIONEER: Peptide InnOvatioN for the Early diabEtes tReatment) semaglutide and are being studied in further trials and registries, including real world data studies. In the current review we discuss the occurrence of adverse events associated with semaglutide focusing on hypoglycemia, gastrointestinal side effects, pancreatic safety (pancreatitis and pancreatic cancer), thyroid cancer, gallbladder events, cardiovascular aspects, acute kidney injury, diabetic retinopathy (DRP) complications and injection-site and allergic reactions and where available, we highlight potential underlying mechanisms. Furthermore, we discuss whether effects are specific for semaglutide or a class effect. We conclude that semaglutide induces mostly mild-to-moderate and transient gastrointestinal disturbances and increases the risk of biliary disease (cholelithiasis). No unexpected safety issues have arisen to date, and the established safety profile for semaglutide is similar to that of other GLP-1RAs where definitive conclusions for pancreatic and thyroid cancer cannot be drawn at this point due to low incidence of these conditions. Due to its potent glucose-lowering effect, patients at risk for deterioration of existing DRP should be carefully monitored if treated with semaglutide, particularly if also treated with insulin. Given the beneficial metabolic and cardiovascular actions of semaglutide, and the low risk for severe adverse events, semaglutide has an overall favorable risk/benefit profile for patient with type 2 diabetes.

## Introduction

With an alarming increase in type 2 diabetes (T2D) prevalence as well as its associated complications (1), the need for adequate treatment strategies for this devastating disease has never been higher. However, apart from studying the potential beneficial effects of new glucose-lowering agents, regulators and clinicians are increasingly focusing on long-term safety aspects. One of the newer antihyperglycemic drug classes receiving such scrutiny on safety are the glucagon-like peptide-1 (GLP-1) receptor agonists (GLP-1RAs). These agents are based on the gut-derived incretin hormone GLP-1, which is a potent stimulator of insulin, while suppressing glucagon secretion (2). In combination with inhibiting effects on gastric emptying and hepatic gluconeogenesis (3), GLP-1RA effectively reduce glucose levels (4). Several agents are now available after the first agent received marketing approval in 2005. Within the class of GLP-1RAs, substantial differences exist in drug structure, efficacy, dosing interval and even adverse effects (5). Nevertheless, in general, a decrease in HbA_1c_ of 1–1.5% is observed, as well as beneficial effects on body weight, blood pressure and lipid profile (4). However, partly due to the widespread presence of GLP-1 receptors, several adverse effects have been observed, of which pancreatitis, pancreatic cancer and thyroid cancer were initially flagged as safety alerts (6).

The most recently approved GLP-1RAs is semaglutide. This agent is somewhat special among GLP-1RAs given that it is the only drug available as both subcutaneous injection (similar to all other GLP-1RAs) and as an oral formulation. Moreover, with years of development after marketing approval of the first GLP-1RA, the registration trials with semaglutide could focus on the already known potential safety risks of this drug class. In this review, as part of a supplement on semaglutide, we will detail the safety aspects of this drug.

## Semaglutide

Semaglutide has been developed based on the vast body of research behind the development of liraglutide (7). Compared to liraglutide, which is administered once daily, semaglutide has an even longer half-life, allowing for once weekly administration. While a significant improvement over once or twice daily subcutaneous administration, the injecting route could be a barrier for some potential users. An absorption enhancer was discovered (sodium *N*-[8-(2-hydroxybenzoyl) aminocaprylate] or SNAC), which, when co-administered with semaglutide, was demonstrated to give therapeutic levels of the latter (8). SNAC helps to protect semaglutide from proteolytic degradation in the stomach and facilitates its absorption across the gastric mucosa by transient effects on transcellular pathways (8). At equivalent levels of exposure, similar glycemic and weight responses have been seen with both oral and subcutaneous semaglutide (9).

Both the subcutaneous and oral formulations of semaglutide have undergone extensive phase 3 clinical testing (**Table 1**). For the once-weekly subcutaneous formulation, the SUSTAIN program (Semaglutide Unabated Sustainability in Treatment of Type 2 Diabetes) included 13 separate randomized clinical phase 3a and 3b trials (10–13, 22, 25–32) SUSTAIN 1 through 10 were global international trials, while three additional trials were specific for China and Japan. In four studies, semaglutide was compared with placebo, with differing patient populations. SUSTAIN-6 is the cardiovascular outcome trial (CVOT) of subcutaneous semaglutide (28).

**Table 1:** Overview of Phase 3 studies of oral semaglutide (PIONEER) and subcutaneous semaglutide (SUSTAIN) (10–32).

Trial	Treatment arms		Key inclusion criteria	Trial duration; blinded or open‐label	Primary endpoint/outcome	Key baseline characteristics (mean values)	Trial product discontinuation/ rescue medication use (proportion of patients)
**PIONEER 1**	Oral semaglutide 3 mg	n=175	Treated with diet and exercise, HbA_1c_ 7.0–9.5%	26‐week; blinded	Change in HbA_1c_ from baseline to week 26	Age: 55 years, HbA_1c_: 8.0% (63 mmol/mol), duration of T2D: 3.5 years	3% / 7%
	Oral semaglutide 7 mg	n=175					8% / 2%
	Oral semaglutide 14 mg	n=175					7% / 1%
	Placebo	n=178					5% / 15%
**PIONEER 2**	Oral semaglutide 14 mg	n=410	Treated with metformin, HbA_1c_ 7.0–10.5%	52‐week; open‐label	Change in HbA_1c_ from baseline to week 26	Age: 58 years, HbA_1c_: 8.1% (65 mmol/mol), duration of T2D: 7.4 years	18% / 8%
	Empagliflozin 25 mg	n=409					11% / 11%
**PIONEER 3**	Oral semaglutide 3 mg	n=466	Treated with metformin ± sulfonylurea, HbA_1c_ 7.0–10.5%	78‐week; blinded	Change in HbA_1c_ from baseline to week 26	Age: 58 years, HbA_1c_: 8.3% (67 mmol/mol), duration of T2D: 8.6 years	17% / 34%
	Oral semaglutide 7 mg	n=464					15% / 22%
	Oral semaglutide 14 mg	n=465					19% / 10%
	Sitagliptin 100 mg	n=466					13% / 28%
**PIONEER 4**	Oral semaglutide 14 mg	n=285	Treated with metformin ± SGLT2i, HbA_1c_ 7.0–9.5%	52‐week; blinded	Change in HbA_1c_ from baseline to week 26	Age: 56 years, HbA_1c_: 8.0% (64 mmol/mol), duration of T2D: 7.6 years	15% / 7%
	Liraglutide 1.8 mg (s.c.)	n=284					13% / 6%
	Placebo	n=142					12% / 30%
**PIONEER 5**	Oral semaglutide 14 mg	n=163	Moderate renal impairment, treated withmetformin ± sulfonylurea; or basal insulin ± metformin, HbA_1c_ 7.0%‐9.5%	26‐week; blinded	Change in HbA_1c_ from baseline to week 26	Age: 70 years, HbA_1c_: 8.0% (64 mmol/mol), duration of T2D: 14.0 years	18% / 4%
	Placebo	n=161					12% / 10%
**PIONEER 6 (CVOT)**	Oral semaglutide 14 mg	n=1591	Age ≥50 years with CVD/CKD or age ≥60 years with CV risk factors	Event‐driven; blinded	3‐point composite MACE	Age: 66 years, HbA_1c_: 8.2% (66 mmol/mol), duration of T2D: 14.9 years	15% / NR
	Placebo	n=1592					10% / NR
**PIONEER 7**	Oral semaglutide (flexible 3, 7 or 14 mg)	n=253	Treated with 1–2 OADs, HbA_1c_ 7.5–9.5%	52‐week; open‐label	Proportion of patients with HbA_1c_ <7.0% at week 52	Age: 57 years, HbA_1c_: 8.3% (67 mmol/mol), duration of T2D: 8.8 years	17% / 3%
	Sitagliptin 100 mg	n=251					9% / 16%
**PIONEER 8**	Oral semaglutide 3 mg	n=184	Treated with insulin ± metformin, HbA_1c_ 7.0–9.5%	52‐week; blinded	Change in HbA_1c_ from baseline to week 26	Age: 61 years, HbA1c: 8.2% (66 mmol/mol), duration of T2D: 15.0 years	13% / 29%
	Oral semaglutide 7 mg	n=181					19% / 18%
	Oral semaglutide 14 mg	n=181					20% / 17%
	Placebo	n=184					12% / 36%
**PIONEER 9**	Oral semaglutide 3 mg	n=49	Treated with diet and exercise or stable dose of 1 OAD, HbA_1c_ 7.0–10.0% if on diet and exercise or HbA_1c_ 6.5–9.5% if on 1 OAD	52‐week; open‐label	Change in HbA_1c_ from baseline to week 26	Age: 59 years, HbA_1c_: 8.2% (66 mmol/mol), duration of T2D: 7.6 years	8% / 14%
	Oral semaglutide 7 mg	n=49					2% / 10%
	Oral semaglutide 14 mg	n=48					6% / 8%
	Liraglutide 0.9 mg (s.c.)	n=48					8% / 6%
	Placebo	n=49					0% / 31%
**PIONEER 10**	Oral semaglutide 3 mg	n=131	Treated with stable doses of 1 OAD, HbA_1c_ 7.0–10.5%	52‐week; open‐label	Number of treatment‐emergent adverse events at week 57	Age: 58 years, HbA_1c_: 8.3% (67 mmol/mol), duration of T2D: 9.4 years	5% / 17%
	Oral semaglutide 7 mg	n=132					7% / 6%
	Oral semaglutide 14 mg	n=130					12% / 2%
	Dulaglutide 0.75 mg (s.c.)	n=65					6% / 9%
**SUSTAIN 1**	S.c. semaglutide 0.5 mg	n=128	Treated with diet and exercise, HbA_1c_ 7.0–10%	30-week; blinded	Change in HbA_1c_ from baseline to week 30	Age: 54 years, HbA_1c_: 8,1% (65 mmol/mol), duration of T2D: 4.2 years	13% / 5%
	S.c. semaglutide 1 mg	n=130					12% / 5%
	Placebo	n=129					11% / 21%
**SUSTAIN 2**	S.c. semaglutide 0.5 mg	n=409	Treated with metformin ± thiazolidinediones, HbA_1c_ 7.0–10.5%	56-week; blinded	Change in HbA_1c_ from baseline to week 56	Age: 55 years, HbA_1c_: 8.1% (65 mmol/mol), duration of T2D: 6.6 years	6% / 5%
	S.c. semaglutide 1 mg	n=409					5% / 2%
	Sitagliptin 100 mg	n=407					5% / 20%
**SUSTAIN 3**	S.c. semaglutide 1 mg	n=404	Treated with 1‐2 OADs, HbA_1c_ 7–10.5%	56-week; open-label	Change in HbA_1c_ from baseline to week 56	Age: 57 years, HbA_1c_: 8.3 (68 mmol/mol), duration of T2D: 9.2 years	20% / 7%
	Exenatide ER 2.0 mg	n=405					21 % / 12%
**SUSTAIN 4**	S.c. semaglutide 0.5 mg	n=362	Treated with metformin ± sulfonylurea, HbA_1c_ 7.0–10.0%	30-week; open-label	Change in HbA_1c_ from baseline to week 30	Age: 57 years, HbA_1c_: 8.2 (66 mmol/mol), duration of T2D: 8.6 years	14% / 17%
	S.c. semaglutide 1 mg	n=360					16% / 18%
	Insulin glargine	n=360					9% / 9%
**SUSTAIN 5**	S.c. semaglutide 0.5 mg	n=132	Treated with insulin ± metformin, HbA_1c_ 7.0–10.0%	30-week; blinded	Change in HbA_1c_ from baseline to week 30	Age: 59 years, HbA_1c_: 8.4% (68 mmol/mol), duration of T2D: 13.3 years	11% / 2%
	S.c. semaglutide 1 mg	n=131					13% / <1%
	Placebo	n=133					10% / 14%
**SUSTAIN 6 (CVOT)**	S.c. semaglutide 0.5 mg	n=826	Age ≥50 years with CVD/CKD or age ≥60 years with CV risk factors	Duration (104-week) and event-driven; blinded	3‐point composite MACE	Age: 65 years, HbA_1c_: 8.7%, duration of T2D: 13.9 years	19.9% / NR
	S.c. semaglutide 1 mg	n=822					22.6% / NR
	Placebo	n=1649					18.8% / NR
SUSTAIN 7*	S.c. semaglutide 0.5 mg	n=301	Treated with metformin, HbA_1c_ 7.0–10.5%	10-week; open-label	Change in HbA_1c_ from baseline to week 40	Age: 66 years, HbA_1c_: 8.2% (66 mmol/mol), duration of T2D: 7.4 years	16% / 1%
	S.c. semaglutide 1 mg	n=300					17% / 2%
	Dulaglutide 0.75 mg (s.c.)	n=299					9% / 5%
	Dulaglutide 1.5 mg (s.c.)	n=299					12% / 2%
SUSTAIN 8*	S.c. semaglutide 1 mg	n=394	Treated with metformin, HbA_1c_ 7.0–10.5%	52-week; blinded	Change in HbA_1c_ from baseline to week 52	Age: 57 years, HbA_1c_: 8.3% (67 mmol/mol), duration of T2D: 7.4 years	16% / 7%
	Canagliflozin 300 mg	n=394					13% / 7%
SUSTAIN 9*	S.c. semaglutide 1 mg	n=151	Treated with metformin ± SGLT2i, HbA_1c_ 7.0–10%	30-week; blinded	Change in HbA_1c_ from baseline to week 30	Age: 57 years, HbA_1c_: 8.0% (64 mmol/mol), duration of T2D: 9.7 years	15% / 0.7%
	Placebo	n=151					8% / 5.3%
SUSTAIN 10*	S.c. semaglutide 1 mg	n=290	Treated with 1–3 OADs, HbA_1c_ 7.0–11.0%	30-week; blinded	Change in HbA_1c_ from baseline to week 30	Age: 60 years, HbA_1c_: 8.2%, duration of T2D: 9.3 years	14.1% / 1.4%
	liraglutide 1.2 mg (s.c.)	n=287					9.1% / 4.2%
**SUSTAIN JAPAN 'Sitagliptin'**	S.c. semaglutide 0.5 mg	n=103	Treated with diet and exercise with HbA_1c_ 7.0–10.5%, or OAD monotherapy with HbA_1c_ 6.5–9.5%	30-week; open-label	Number of treatment‐emergent adverse events at week 30	Age: 58 years, HbA_1c_: 8.1%, duration of T2D: 8.0 years	2.9% / 0.9%
	S.c. semaglutide 1 mg	n=102					14.7% / 0
	Sitagliptin 100 mg	n=103					2.9% / 4.9%
**SUSTAIN JAPAN 'individual'**	S.c. semaglutide 0.5 mg	n=239	Treated with diet and exercise, or OAD monotherapy, HbA_1c_ 7.0–10.5%	56-week; open-label	Number of treatment‐emergent adverse events at week 56	Age: 59 years, HbA_1c_: 8.1% (65 mmol/mol), duration of T2D: 8.8 years	6.3% / 0%
	S.c. semaglutide 1 mg	n=241					14.1% / 0.4%
	Additional OAD (investigators discretion)	n=120					5.9% / 6.7%
**SUSTAIN China**	S.c. semaglutide 0.5 mg	n=287	Treated with metformin, HbA_1c_ 7.0–10.5%	30-week; blinded	Change in HbA_1c_ from baseline to week 30	Age: 53 years, HbA1c 8.1%, Duration of T2D: 6.4 years	NR / 3.1%
	S.c. semaglutide 1 mg	n=290					NR / 1.4%
	Sitagliptin 100 mg	n=290					NR / 6.6%

*Phase 3b trials all others are phase 3a trials CKD, chronic kidney disease; CV, cardiovascular; CVOT, cardiovascular outcomes trial; CVD, cardiovascular disease; ER, extended release; MACE, major adverse cardiovascular event; NR, not reported; OAD, oral antidiabetic drug; s.c, subcutaneous; SGLT2i, sodium-glucose co-transporter-2 inhibitor; T2D, type 2 diabetes.

The PIONEER program (Peptide InnOvatioN for the Early diabEtes tReatment) comprised 10 individual trials comparing once-daily oral semaglutide with placebo (six studies) or active comparator in different populations (14–21, 23, 24). Similar to the SUSTAIN program, PIONEER 6, was the CVOT (19). PIONEER 9 and 10 are specific to the Japanese population (12, 13). The SOUL (A Heart Disease Study of Semaglutide in Patients with Type 2 Diabetes) study is a larger CVOT with oral semaglutide that is currently ongoing (NCT03914326).

Combining all individual studies, the SUSTAIN program contained almost 12,000 participants, with over 9,500 subjects in the PIONEER program. With treatment duration of at least 26 weeks, this accounts of many patient years of follow-up, allowing an adequate review of the safety of semaglutide.

## Adverse Effects of Semaglutide

**Figure 1:**
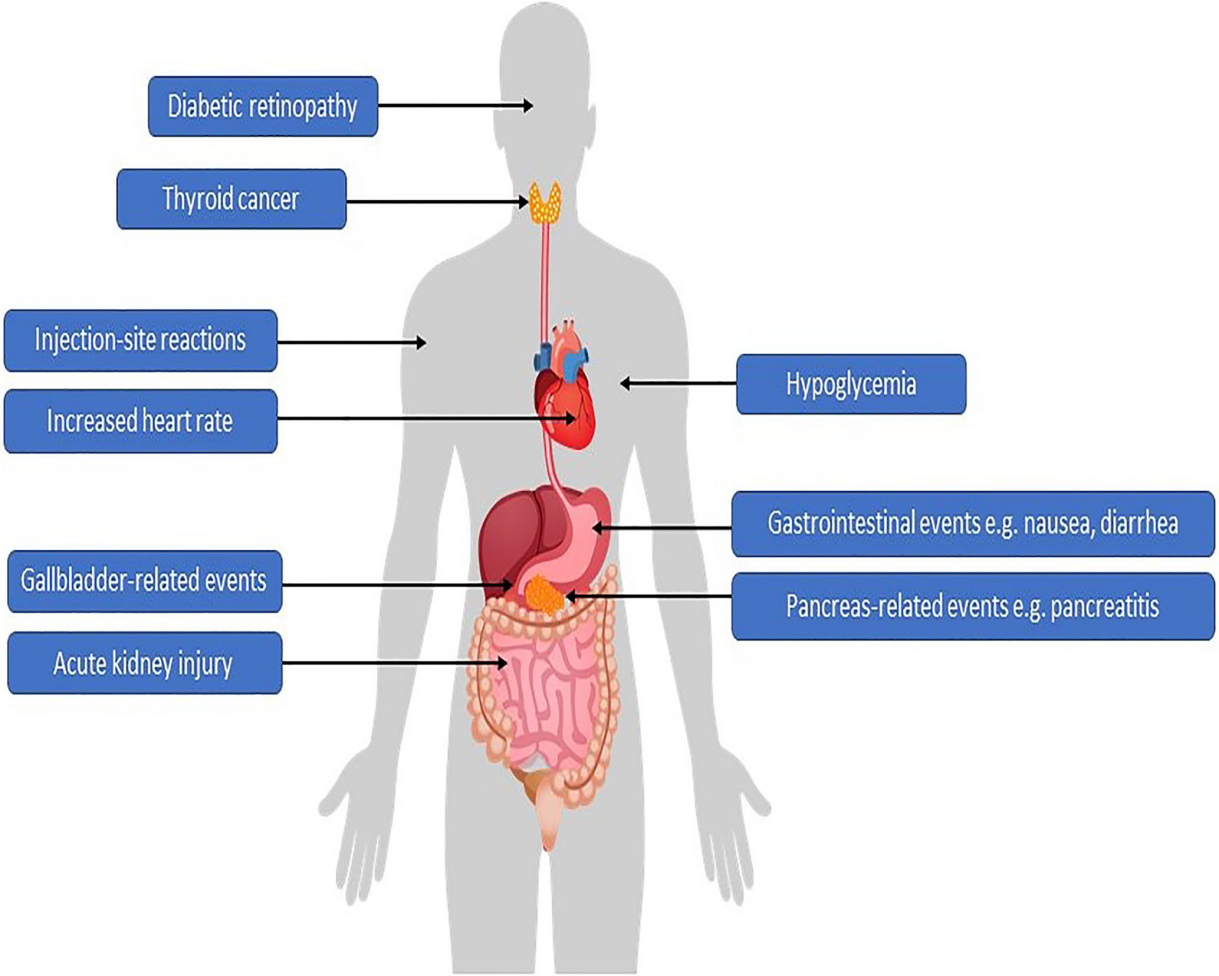
Potential adverse effects associated with GLP1-RAs.

Semantically, the on-target effects of GLP-1RAs are those effects leading to a reduction in glucose levels. Any other effect can be considered as a pleiotropic, off-target effect, or in the case of unwanted actions, adverse effects (**Figure 1**). Many of the (adverse) class effects are shared among the different GLP-1RA, however, differences do occur. For semaglutide, one could expect a different side-effect profile for the oral *versus* the subcutaneous formulation. Apart from the obvious—tablets will not induce injection-site reactions—it could be suggested that higher portal levels induce more gastrointestinal disturbances. Moreover, with the maximum oral dosage plasma levels are lower compared with the maximal subcutaneous dose (oral 20 mg yields plasma levels of ~25 nM, subcutaneous 1 mg yields plasma levels of ~45 nM (33, 34)). Worth noting is that no data comparing the pharmacokinetic profile of both formulations against each other are available. In the following sections, the adverse reactions and safety issues of semaglutide, both oral and subcutaneous, will be discussed. We will discuss the risk of hypoglycemia, gastrointestinal side effects including previous reports on increased risk for pancreatitis and pancreas cancer, thyroid cancer, gallbladder stones, effects on the cardiovascular system, acute kidney injury, diabetic retinopathy risks and allergies/injection-site reactions (**Table 2**).

**Table 2:** Adverse effects and safety risks in phase 3 trials (10–32).

Treatment arms	Incidence of AE, n (%)											% of patients with AE leading to trial product discontinuation	
	Any	Severe or confirmed symptomatic hypoglycemic episode*	Gastrointestinal			Pancreas		Gallbladder	Thyroid	Diabetic retinopathy	Acute kidney Injury	Any AE, n(%)	Gastrointestinal, %
			Nausea	Vomiting	Diarrhea	Pancreatitis	Pancreatic cancer						
**PIONEER 1**													
Oral semaglutide 3 mg	101 (57.7)	5 (2.9)	14 (8.0)	5 (2.9)	15 (8.6)	0		NR	0	1 (0.6%)	0	4 (2.3)	75
Oral semaglutide 7 mg	93 (53.1)	2 (1.1)	9 (5.1)	8 (4.6)	9 (5.1)	0		NR	0	6 (3.4%)	0	7 (4.0)	57
Oral semaglutide 14 mg	99 (56.6)	1 (0.6)	28 (16.0)	12 (6.9)	9 (5.1)	0		NR	0	2 (1.1%)	1 (0.6)	13 (7.4)	69
Placebo	99 (55.6)	1 (0.6)	10 (5.6)	4 (2.2)	4 (2.2)	0		NR	0	3 (1.7%)	1 (0.6)	4 (2.2)	25
**PIONEER 2**													
Oral semaglutide 14 mg	289 (70.5)	7 (1.7)	81 (19.8)	30 (7.3)	38 (9.3)	1 (0.2)	0	NR	0	14 (3.4)	2 (0.5)	44 (10.7)	75
Empagliflozin 25 mg	283 (69.2)	8 (2.0)	10 (2.4)	7 (1.7)	13 (3.2)	1 (0.2)	0	NR	0	5 (1.2%)	1 (0.2)	18 (4.4)	17
**PIONEER 3**													
Oral semaglutide 3 mg	370 (79.4)	23 (4.9)	34 (7.3)	13 (2.8)	45 (9.7)	1 (0.2)	0	NR	0	27 (5.8)	3 (0.6)	26 (5.6)	42
Oral semaglutide 7 mg	363 (78.2)	24 (5.2)	62 (13.4)	28 (6.0)	53 (11.4)	1 (0.2)	0	NR	0	24 (5.2)	2 (0.4)	27 (5.8)	56
Oral semaglutide 14 mg	370 (79.6)	36 (7.7)	70 (15.1)	42 (9.0)	57 (12.3)	1 (0.2)	1 (0.2)	NR	0	16 (3.4)	5 (1.1)	54 (11.6)	59
Sitagliptin 100 mg	388 (83.3)	39 (8.4)	32 (6.9)	19 (4.1)	37 (7.9)	1 (0.2)	1 (0.2)	NR	0	27 (5.8)	3 (0.6)	24 (5.2)	50
**PIONEER 4**													
Oral semaglutide 14 mg	229 (80)	2 (1)	56 (20)	25 (9)	43 (15)	0	0	NR	1 (0.4)	8 (3)	0	31 (11)	71
Liraglutide 1.8 mg (s.c.)	211 (74)	7 (2)	51 (18)	13 (5)	31 (11)	1 (0.4)	1 (0.4)	NR	1 (0.4)	4 (1)	1 (0.4)	26 (9)	65
Placebo	95 (67)	3 (2)	5 (4)	3 (2)	11 (8)	1 (0.7)	0	NR	0	2 (1)	1	5 (4)	60
**PIONEER 5**													
Oral semaglutide 14 mg	122 (75)	9 (6)	31 (19)	19 (12)	17 (10)	0	0	NR	0	5 (3)	3 (1.8)	24 (15)	79
Placebo	109 (68)	3 (2)	12 (7)	6 (4)	6 (4)	0	0	NR	0	2 (1)	1 (0.6)	8 (5)	38
**PIONEER 6**													
Oral semaglutide 14 mg	NR	NR	NR	NR	NR	1 (0.1)	0	NR	2 (0.1)	93 (5.8)	32 (2.0)	184 (11.6)	59
Placebo	NR	NR	NR	NR	NR	3 (0.2)	0	NR	0	76 (4.8)	37 (2.3)	104 (6.5)	25
**PIONEER 7**													
Oral semaglutide (flexible 3, 7 or 14 mg)	197 (78)	14 (5.5)	53 (21)	14 (6)	22 (9)	0	0	NR	0	6 (2.4)	1 (0.4)	22 (9)	64
Sitagliptin 100 mg	172 (69)	14 (5.6)	6 (2)	3 (1)	8 (3)	0	0	NR	0	6 (2.4)	0	8 (3)	25
**PIONEER 8**													
Oral semaglutide 3 mg	137 (74.5)	52 (28.3)	21 (11.4)	11 (6.0)	16 (8.7)	0	0	NR	0	7 (3.8)	2 (1.1)	13 (7.1)	69
Oral semaglutide 7 mg	142 (78.5)	47 (26.0)	30 (16.6)	14 (7.7)	22 (12.2)	0	0	NR	0	8 (4.4)	1 (0.6)	16 (8.8)	75
Oral semaglutide 14 mg	151 (83.4)	48 (26.5)	42 (23.2)	18 (9.9)	27 (14.9)	0	0	NR	0	9 (5.0)	0	24 (13.3)	79
Placebo	139 (75.5)	54 (29.3)	13 (7.1)	7 (3.8)	11 (6.0)	0	0	NR	0	8 (4.3)	0	5 (2.7)	20
**PIONEER 9**													
Oral semaglutide 3 mg	37 (76)	0	2 (4)	NR	4 (8)	0	0	NR	0	0	0	1 (2)	100
Oral semaglutide 7 mg	37 (76)	0	5 (10)	NR	1 (2)	0	0	NR	1	1 (2.0)	0	1 (2)	100
Oral semaglutide 14 mg	34 (71)	0	4 (8)	NR	3 (6)	0	0	NR	0	1 (2.1)	0	2 (4)	100
Liraglutide 0.9 mg (s.c.)	32 (67)	2 (4.2)	0	NR	2 (4)	0	0	NR	0	0	0	0	0
Placebo	39 (80)	0	1 (2)	NR	1 (2)	0	0	NR	0	2 (4.1)	0	0	0
**PIONEER 10**													
Oral semaglutide 3 mg	101 (77)	3 (2)	7 (5)	3 (2)	2 (2)	0	0	2 (2)	0	9 (7)	0	4 (3)	50
Oral semaglutide 7 mg	106 (80)	3 (2)	11 (8)	6 (5)	2 (2)	0	0	1 (1)	0	12 (9)	0	8 (6)	50
Oral semaglutide 14 mg	111 (85)	4 (3)	12 (9)	9 (7)	10 (8)	0	0	0	0	7 (5)	0	8 (6)	63
Dulaglutide 0.75 mg (s.c.)	53 (82)	0	6 (9)	1 (2)	4 (6)	0	0	1 (2)	0	3 (5)	0	2 (3)	50
**SUSTAIN 1**													
S.c. semaglutide 0.5 mg	82 (64)	0	26 (20)	5 (4)	16 (13)	0	0	3 (2)	0	NR	0	8 (6)	63
S.c. semaglutide 1 mg	73 (56)	0	31 (24)	9 (7)	14 (11)	0	0	1 (<1)	0	NR	0	7 (5)	57
Placebo	69 (53)	3 (2)	10 (8)	2 (2)	3 (2)	0	0	0	0	NR	0	3 (2)	33
**SUSTAIN 2**													
S.c. semaglutide 0.5 mg	306 (75)	7 (2)	73 (18)	33 (8)	54 (13)	3 (1%)	NR	1 (<1)	0	1 (<1)	NR	33 (8)	82
S.c. semaglutide 1 mg	292 (71)	2 (<1)	72 (18)	41 (10)	53 (13)	1 (<1)	NR	7 (2)	1	0	NR	39 (10)	79
Sitagliptin 100	292 (72)	5 (1)	30 (7)	11 (3)	29 (7)	0	NR	6 (1)	0	3 (1)	NR	12 (3)	25
**SUSTAIN 3**													
S.c. semaglutide 1 mg	303 (75)	33 (8.2)	90 (22.3)	29 (7.2)	46 (11.4)	2 (<1)	NR	6 (1%)	NR	NR	NR	38 (9.4)	NR
Exenatide ER 2.0 mg	309 (76.3)	33 (8.1)	48 (11.9)	25 (6.2)	34 (8.4)	3 (<1)	NR	2 (<1)	NR	NR	NR	29 (7.2)	NR
**SUSTAIN 4**													
S.c. semaglutide 0.5 mg	253 (70)	16 (4)	77 (21)	24 (7)	59 (16)	2 (1)	1 (<1)	1 (<1)	NR	1 (<1)	NR	20 (6)	55
S.c. semaglutide 1 mg	264 (73)	20 (6)	80 (22)	37 (10)	69 (19)	0	0	2 (1)	NR	0	NR	27 (8)	70
Insulin glargine	235 (65)	38 (11)	13 (4)	11 (3)	16 (4)	0	0	0	NR	1 (<1)	NR	4 (1)	0
**SUSTAIN 5**													
S.c. semaglutide 0.5 mg	91 (68.9)	11 (8.3)	15 (11.4)	8 (6.1)	6 (4.5)	0	0	3 (2.3)	0	(3.0)	NR	6 (4.5)	NR
S.c. semaglutide 1 mg	84 (64.1)	14 (10.7)	22 (16.8)	15 (11.5)	9 (6.9)	0	0	1 (0.8)	0	(0.8)	NR	8 (6.1)	NR
Placebo	77 (57.9)	7 (5.3)	6 (4.5)	4 (3.0)	2 (1.5)	0	0	0	0	0	NR	1 (0.8)	NR
**SUSTAIN 6**													
S.c. semaglutide 0.5 mg	740 (89.6)	191 (23.1)	143 (17.3)	14 (1.7)	15 (1.8)	6 (0.7)	0	25 (3)	0		42 (5.1)	95 (11.5)	49
S.c. semaglutide 1 mg	732 (89.1)	178 (21.7)	180 (21.9)	23 (2.8)	19 (2.3)	3 (0.4)	1 (0.1)	17 (2.1)	0	50 (3.0)	23 (2.8)	119 (14.5)	65
Placebo	1484 (90)	350 (21.2)	129 (7.8)	5 (0.3)	7 (0.4)	12 (0.7)	4 (0.2)	39 (2.3)	0	29 (1.8)	34 (4.1)	110 (6.7)	16
**SUSTAIN 7**													
S.c. semaglutide 0.5 mg	204 (68)	2 (1)	68 (23)	31 (10)	43 (14)	0	0	2 (1)	1 (<1)	2 (1)	NR	24 (8)	67
S.c. semaglutide 1 mg	207 (69)	5 (2)	63 (21)	31 (10)	41 (14)	0	0	4 (1)	0	2 (1)	NR	29 (10)	62
Dulaglutide 0.75 mg (s.c.)	186 (62)	3 (1)	39 (13)	12 (4)	23 (8)	0	0	4 (1)	0	2 (1)	NR	14 (5)	43
Dulaglutide 1.5 mg (s.c.)	221 (74)	5 (2)	60 (20)	29 (10)	53 (18)	0	0	8 (3)	1 (<1)	3 (1)	NR	20 (7)	70
**SUSTAIN 8**													
S.c. semaglutide 1 mg	298 (76)	53 (14)	89 (23)	50 (13)	60 (15)	NR	NR	NR	NR	9 (2)	4 (1)	38 (10)	68
Canagliflozin 300 mg	283 (72)	32 (8)	26 (7)	9 (2)	37 (9)	NR	NR	NR	NR	15 (4)	0	20 (5)	20
**SUSTAIN 9**													
S.c. semaglutide 1 mg	104 (69.3)	17 (11.3)	29 (19.3)	14 (9.3)	17 (11.3)	0	0	NR	NR	3 (2.0)	1 (0.7)	13 (8.7)	77
Placebo	91 (60.3)	3 (2.0)	5 (3.3)	3 (2.0)	9 (6.0)	0	0	NR	NR	8 (5.3)	0	3 (2.0)	0
**SUSTAIN 10**													
S.c. semaglutide 1 mg	204 (70.6)	5 (1.7)	63 (21.8)	30 (10.4)	45 (15.6)	0	NR	NR	NR	3 (1.0)	NR	33 (11.4)	67
Liraglutide 1.2 mg (s.c.)	190 (66.2)	7 (2.4)	45 (15.7)	23 (8.0)	35 (12.2)	2 (0.7%)	NR	NR	NR	4 (1.4)	NR	19 (6.6)	58
**SUSTAIN JAPAN 'SITA'**													
S.c. semaglutide 0.5 mg	77 (74.8)	0	(10.7)		(6.8%)	0	0	1 (1.0)	0	4 (3.9)	NR	3 (2.9)	NR
S.c. semaglutide 1 mg	73 (71.6)	1 (1.0)	(12.7)		(8.8%)	0	0	3 (2.9)	0	2 (1.9)	NR	11 (10.8)	NR
Sitagliptin 100 mg	68 (66.0)	0	0		(1.9%)	0	1 (1.0)	0	0	4 (3.9)	NR	2 (1.9)	NR
**SUSTAIN JAPAN 'INDIVIDUAL'**													
S.c. semaglutide 0.5 mg	206 (86.2)	3 (1.3)	29 (12.1)	13 (5.4)	24 (10.0)	0	0	4 (1.7%)	0	11 (4.6)	NR	14 (5.9)	NR
S.c. semaglutide 1 mg	212 (88)	6 (2.5)	46 (19.1)	14 (5.8)	38 (15.8)	0	0	2 (0.8%)	0	16 (6.6)	NR	26 (10.8)	NR
Additional OAD (investigators discretion)	86 (71.7)	2 (1.7)	1 (0.8)	2 (1.7)	8 (6.7)	0	0	0	0	6 (5.0)	NR	4 (3.3)	NR
**SUSTAIN China**													
S.c. semaglutide 0.5 mg	209 (72.8%)	2 (0.7%)	22 (7.7%)	14 (4.9%)	58 (20.2%)	0	0	NR	NR	19 (6.6%)	NR	17 (5.9%)	59
S.c. semaglutide 1 mg	216 (74,5%)	6 (2.1%)	39 (13.4%)	19 (6.6%)	49 (16.9%)	1 (0.3%)	0	NR	NR	14 (4.8%)	NR	31 (10.7%)	68
Sitagliptin 100 mg	199 (68,6%)	4 (1.4%)	5 (1.7%)	3 (1.0%)	20 (6.9%)	0	0	NR	NR	10 (3.4%)	NR	6 (2.1%)	17

AE, adverse event; ER, extended release; NR, not reported; OAD, oral antidiabetic drug; s.c. subcutaneous.

An independent external adjudication committee (EAC) validated prespecified categories of adverse events (including deaths, selected cardiovascular events, malignant neoplasms, thyroid diseases [malignant thyroid neoplasms and C-cell hyperplasia], acute kidney injury, acute pancreatitis, and lactic acidosis) except in SUSTAIN 10 where there was no adjudication.

*An episode that was severe according to the ADA classification (requires assistance of another person to actively administer carbohydrate, glucagon, or other corrective action) or an episode with confirmed blood glucose value <56 mg/dL and symptoms consistent with hypoglycemia.

### Hypoglycemia

Given that the aim of GLP-1RA therapy is mainly to reduce blood glucose levels, it is conceivable that these agents could cause hypoglycemia. However, since GLP-1RA mainly lower blood glucose by stimulating glucose-dependent insulin secretion, hypoglycemia is an infrequent problem. In addition, the inhibition of glucagon release does not occur under hypoglycemic conditions (35). In SUSTAIN-6, severe or plasma glucose-confirmed (<56 mg/dl [3.1 mol/L]) hypoglycemia occurred in similar rates between patients with semaglutide (23.1% in the 0.5 mg group and 21.7% in the 1 mg group) and placebo (21.2%) (28). In comparison, in SUSTAIN-4, severe or confirmed hypoglycemia occurred in 11% of insulin glargine-treated patients, compared with 4–6% in the semaglutide-treated patients (26). Importantly, in SUSTAIN-4 it is reported that hypoglycemia predominantly occurred in subjects using sulfonylurea agents (26). To illustrate: in the group of subjects randomized to semaglutide 1 mg, 9% of subjects using a s sulfonylurea had a severe or blood-glucose confirmed hypoglycemia, *versus* 2% in those not using a sulfonylurea. Similarly, in SUSTAIN-3, the majority of hypoglycemic events were reported in subjects concomitantly receiving sulfonylureas in both the semaglutide 1.0 mg and exenatide ER 2.0 mg groups. For oral semaglutide, the percentage of patients with severe hypoglycemia was 1.4% with oral semaglutide and 0.8% with placebo in PIONEER 6 (19). Here all severe hypoglycemic events occurred in patients receiving concomitant insulin or sulfonylurea therapy at the time of the event. In other phase 3 trials, no increase in hypoglycemia risk was observed *versus* comparator groups, including other GLP-1RAs (18–21, 23, 33–36).

Real world data with respect to hypoglycemia are limited to a single observational cohort from Canada (36). In 815 individuals who started semaglutide therapy and were followed for 6 months, there was no change in overall reported hypoglycemia. Although the group of concomitant insulin users also reported no change in hypoglycemia occurrence, this could have been mitigated by the on average 10–20% reduction in total daily insulin dosage (36). Sulfonylurea users did not experience an increase in hypoglycemia events.

Thus, the risk of hypoglycemia appears to be low with subcutaneous and oral semaglutide by themselves, yet the risk is increased when combined with sulfonylurea and/or insulin therapy. Several experts advise to lower the dose of sulfonylurea and short-acting and low-acting insulin analogues prior to or during titration of GLP-1RA therapy, to reduce the risk of (severe) hypoglycemia (37).

### Gastrointestinal (GI) Adverse Effects

In the phase 3 trials, both oral and subcutaneous semaglutide were associated with gastrointestinal disturbances, such as nausea, vomiting and diarrhea, well-known effects from this drug class. When compared with placebo, subcutaneous semaglutide for 30 weeks induced nausea in 11.4 to 20% of the semaglutide-treated patients (placebo 3.3–8%), vomiting in 4 to 11.5% (placebo 2–3%) and diarrhea in 4.5 to 11.3% (placebo 1.5–6%) (10, 27, 31). In SUSTAIN 6, where generally older patients with comorbid conditions were treated for 104 weeks, the incidence of GI disturbances was somewhat higher (28). For oral semaglutide, the placebo-controlled trials found nausea ranged between 5.1 and 23.2% (placebo 5.6–7.1%), vomiting between 2.9 and 9.9% (placebo 2.2–3.8%) and diarrhea between 5.1 and 15% (placebo 2.2–8%) during the on-treatment period (14, 17, 21). These rates were not different when focusing on Japanese patients [PIONEER 9 (23)], but appeared higher in patients with T2D, reduced kidney function (estimated glomerular filtration rate [GFR] of 30–59 ml/min) and comorbidities in PIONEER 5 (18).

In one phase 2 trial, subcutaneous and oral semaglutide were compared with each other (38). Here, patients were randomized to oral semaglutide (at a dose of 5, 10, 20 or 40 mg once daily), subcutaneous semaglutide (1 mg once weekly) or placebo. As discussed below, this study also assessed the effect of dose escalation in two additional groups. Unfortunately, the currently advocated oral treatment doses of 7 and 14 mg were not included. When comparing oral 20 mg to subcutaneous 1 mg, the total amount of gastrointestinal disturbances was similar (56% *versus* 54%, respectively). This was also true for nausea (34% *versus* 32%), vomiting (16% *versus* 9%) and diarrhea (20% *versus* 14%). The proportion of patients with premature discontinuation because of adverse events appeared higher for oral semaglutide 20 mg (27%) than for subcutaneous semaglutide 1 mg (14%). All numbers were similar between the 10 and 20 mg oral dose, except for treatment discontinuation, which was 12% for the lower dosage.

**Figure 2:**
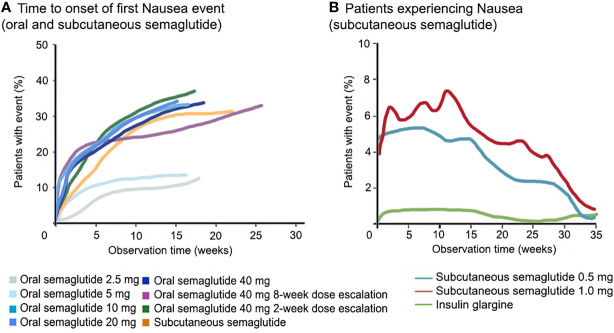
Course of nausea with semaglutide. GLP-1RA, including semaglutide, cause nausea in about one third of treated patients, which is both dose- and time-dependent. In panel **(A)**, a direct comparison between subcutaneous and oral semaglutide is shown, as well as different doses of oral semaglutide, for the first occurrence of nausea. In panel **(B)**, the course of the occurrence of nausea is shown for subcutaneous semaglutide. Data for panel **(A)** are derived from the phase-2 trial (38), for panel **(B)** data are shown from (26). GLP-1RA, glucagon-like peptide-1 receptor agonist.

Importantly, for both formulations, higher doses are often associated with more frequent GI adverse effects. For this reason, a dose escalation scheme is advised, starting with a low dose (3 mg). As a clear example in the abovementioned phase 2 study (38), 77% of patients experienced GI adverse effects when a fast 2-week dose escalation was used to reach 40 mg compared with 54% in the slower 8-week dose-escalation group. Generally, the GI complaints with semaglutide occur in the first 8–12 weeks of treatment during dose escalation [in contrast to for example liraglutide, where they occur within 2 weeks (17, 32)], and wane over time (**Figure 2**). Overall, the adverse effects are mild to moderate in severity and often self-limiting.

Nevertheless, GI complaints are the main adverse-event related cause of drug discontinuation in the phase-3 trials, with rates up to 12% (**Table 2**). Moreover, cohorts with real-world data show similar numbers. In one retrospective study where 189 patients with T2DM starting subcutaneous semaglutide, 9.5% discontinued therapy because of GI complaints, while in 5.8% such adverse effects limited uptitration (39). In another cohort where 164 T2DM patients were switched from a different GLP-1RA therapy to semaglutide, 10.4% discontinued semaglutide because of adverse GI effects (40). Combined, data from clinical trials and clinical practice suggest that approximately 10% of patients will discontinue semaglutide because of GI complaints, which may be a bit higher compared to other GLP-1 analogues.

Apart from gradual dose titration, data on how to prevent or treat GI disturbances with GLP-1RA are limited. Patients can be counseled to eat slowly with reduced portion size per meal, stop eating when they experience satiety, and to avoid high-fat food (41). Anti-emetic therapy has been found effective in healthy subjects (42), but are not common practice since long-term data are not available. Interestingly, in one systematic analysis, background use of metformin was associated with more nausea and vomiting when using a GLP-1RA (43). However, whether this is also true for the combination with semaglutide, or whether lowering the dose of metformin has effect, has not been studied.

The mechanisms behind nausea/vomiting and diarrhea are incompletely understood. For nausea, a relation with the inhibiting effects on gastric emptying seems plausible. However, nausea also occurs in the fasting state (44), and is not related to measures of gastric emptying speed after meal ingestion (45). An effect on the central nervous system has been suggested as a recent study with modified exenatide—with reduced brain penetrance—showed less vomiting in musk shrews, despite retaining effects on glucose control (46). For diarrhea, studies are lacking. In one study, osmotic diarrhea occurred 8 h after infusion of GLP-1 peptide (47), and GLP-1RAs have been shown to reduce intestinal uptake of glucose and lipids (48, 49). Also, in patients with type 1 diabetes, liraglutide reduced colon transit time (50). So, hypothetically, semaglutide could induce diarrhea by altering nutrient absorption or intestinal motility.

Finally, although nausea and vomiting are perhaps unwanted effects, they may also be partly responsible for aspects of the drug’s efficacy as indicated above. As such, in some studies, nausea induced by GLP-1RAs is linked to weight loss (51, 52). For example, obese subjects treated with high-dose liraglutide who experienced (transient) nausea had on average 2.9 kg (95%-CI 0.5–5.3) more weight loss compared to those without GI events (51). In a mediation analysis of the SUSTAIN 1 to 5 trials, a small component (0.07 to 0.5 kg) of the total treatment difference in weight loss was explained by nausea or vomiting (52). In contrast, when combining data from SUSTAIN 3, 7 and 10, the occurrence of nausea and vomiting was not associated with superior weight loss (53). Whether this route plays a role in the beneficial effects of GLP-1RA on body weight needs further studying.

### Pancreatic Adverse Events: Pancreatitis and Pancreatic Cancer

Within years of the introduction of GLP-1RAs, these agents were linked to the occurrence of acute pancreatitis, and suggested to potentially cause pancreatic cancer (6). In the subsequent years, many pharmacovigilance and database studies followed, with conflicting results (54–60). Given the nature of observational studies, data could have been confounded, since patients with diabetes whom have an indication for GLP-1RA therapy often have concomitant risk factors for pancreatitis (notably obesity, longer diabetes duration and co-medication). As such, the longer term CVOTs were a welcome addition to the discussion. When focusing on semaglutide, no signals of pancreatic AEs were present with blinded adjudication. In SUSTAIN 6, acute pancreatitis occurred in 9 semaglutide-treated patients, and in 12 placebo-treated patients. Pancreatic cancer occurred in one and four patients, respectively (28). In PIONEER 6, acute pancreatitis occurred in one semaglutide-treated patient, and in three placebo-treated patients (19). The incidence of pancreatic cancer was not reported. When combining all phase 3a data, pancreatitis occurred in five semaglutide-treated patients in PIONEER (six in the comparator group), and in 15 patients in SUSTAIN (13 in the comparator group). However, it is possible that for a relatively rare complication (the background incidence of pancreatitis and pancreatic cancer in T2D patients is 422 and 15–24 per 100,000 person-years, respectively (61, 62)), the CVOTs and phase 3 studies are of insufficient power to show differences between groups. When combining all available CVOT data (including those from non-semaglutide GLP-1RAs) in a meta-analysis, a hazard ratio of 1.05 (95% confidence interval [CI] 0.78–1.40) was found for pancreatitis and 1.12 (95% CI 0.77–1.63) for pancreatic cancer (63). These data thus argue against an effect of GLP-1RA on pancreatitis and pancreatic cancer incidence. However, one can wonder whether the follow-up duration in the CVOTs (ranging from a median of 1.3 to 5.4 years) is long enough for patients to develop pancreatic cancer.

While establishing (the absence of) a link with pancreatitis and pancreatic cancer in large clinical studies was one aspect in this field of research, others focused on animal studies and more mechanistic findings. One consistent finding is a subtle and asymptomatic increase in plasma lipase and amylase level (64, 65), which occurs within hours of administration (66). In a 26-week randomized controlled trial, oral semaglutide dose-dependently increased lipase levels by 9 to 55% and subcutaneous semaglutide by 36% (38). An increase in enzyme levels was not associated with occurrence of pancreatic events in trials with liraglutide (67, 68). Moreover, our group previously demonstrated that the liraglutide-induced increase in pancreatic enzymes is not associated with changes in pancreatic exocrine function or pancreas size measured by magnetic resonance imaging (69). Such studies have not yet been conducted for semaglutide.

A handful of preclinical studies showed that GLP-1RAs induce pancreatic inflammation, cellular proliferation and intra-epithelial neoplasia (PanIN) (70–72). However, the majority of animal studies did not find any effect of GLP-1RAs on pancreatic physiology, even with doses up to 240 the normal human dose (73–76). Preclinical studies with semaglutide also found no adverse signals in pancreatic tissue (76). Although pancreatic adverse events are difficult to completely rule out, an assessment by the FDA and the EMA concluded that a causal association between incretin-based drugs and pancreatitis or pancreatic cancer is inconsistent with the current data (77).

### Thyroid Cancer

Both formulations of semaglutide have received an official box warning for thyroid C-cell tumors in the US. This caution is solely based on data from rodent studies and is not unique for semaglutide amongst the GLP-1RA. In rodents, the thyroid C-cells (neuroendocrine parafollicular cells which secrete calcitonin) highly express the GLP-1 receptor (78). Stimulation leads to upregulation of the calcitonin gene expression, calcitonin synthesis, C-cell hyperplasia, and increased risk of medullary adenomas and carcinomas (78). Initial studies found expression of the GLP-1 receptor in healthy human thyroid tissue, as well as in medullary thyroid carcinoma (MTC) and C-cell hyperplasia (79, 80). However, these studies were later refuted, as incompletely validated GLP-1 receptor antisera were used (81). When using validated antibodies, the GLP-1 receptor is only marginally expressed in thyroids of non-human primates and humans (78, 82). Supporting this is the observation that monkeys treated with >60 times the human dose of liraglutide do not develop C-cell abnormalities after 20 months (78).

In the SUSTAIN program, three adjudicated events of malignant thyroid neoplasm were identified, two in semaglutide-treated patients (combined n = 5,933), and one in the comparator group (n = 4,736) (10–13, 22, 26–32). None of these were medullary carcinoma. Serum calcitonin was measured during these trials, and no notable difference in mean levels was seen between the treatment arms. In the PIONEER program, four thyroid malignancies occurred in semaglutide-treated patients, *versus* one in the comparator group (14–21, 23, 24). In one instance, a MTC developed in a patient with preexisting nodules and elevated calcitonin at baseline (19). When looking at long-term data from the LEADER trials, the CVOT for liraglutide, there was no difference between liraglutide and placebo regarding calcitonin levels and C-cell malignancies (83).

It should be noted that MTC is rare (estimated incidence of 0.2 cases per 100,000 patient-years), and as such, it is very difficult to definitively rule out an association between GLP-1RA and thyroid malignancies. Therefore, regulatory authorities required additional pharmacovigilance activities, by systematically monitoring the annual incidence of MTC in the US for at least 15 years (MTC-22341, results expected by 2035–2037). In the meantime, semaglutide is contraindicated in patients with a personal or family history of MTC, as well as in patients with multiple endocrine neoplasia (MEN) type 2 in the US.

### Gallbladder

In the SCALE-trial, high-dose liraglutide for the treatment of obesity was associated with an increased risk of gallbladder events compared to placebo (2.5% *versus* 1.0% of patients, respectively) (84). Based on AEs as reported in the European EudraVigilance database, gallbladder disease is likely not limited to liraglutide, but affects all incretin-based therapies (85). A recent meta-analysis observed an increased risk of 28% for cholelithiasis with GLP-1RA treatment (86), but a breakdown for each agent was not given. In the SUSTAIN program, 83 patients (1.4%) treated with semaglutide developed a gallbladder event, compared with 39 patients (1.9%) in the placebo group (10, 27, 28, 31, 32). The events mainly included cholelithiasis. In the PIONEER program, cholelithiasis occurred more often in the semaglutide-treated group (0.6% *versus* 0.1% with placebo), while the risk of cholecystitis was similar (data derived from the summary of product characteristics (SmPC) (87), as the manuscripts did not describe these data). Importantly, none of the gallbladder events have been linked to mortality. Cholelithiasis has been included in the SmPC of both subcutaneous and oral semaglutide).

Initially the gallbladder events were attributed to GLP-1RA-induced weight loss, as for example in the SCALE and LEADER trials, the patients with gallbladder events had more than average weight loss (84, 88). However, as gallbladder disease is not an issue with sodium-glucose co-transporter-2 (SGLT-2) inhibitors (with similar weight loss) (89), and gallbladder events also occurred in GLP-1RA-treated patients well after weight reduction (90), other mechanisms are possibly in play. One option could be lower gallbladder motility, which enhances biliary sludge formation and bile stones. In acute intervention studies, exenatide and albiglutide reduced cholecystokinin-induced gallbladder emptying (91, 92). However, after 12-week liraglutide intervention, we were unable to demonstrate an effect on gallbladder emptying (93), while Nexøe-Larsen et al. observed that liraglutide prolonged the time to reach maximum gallbladder emptying (94). Another mechanism is a change in bile salts, leading to supersaturated bile. While we observed changes in deoxycholic acid levels in plasma and fecal samples after liraglutide treatment, the clinical relevance remains unclear (93). Fascinatingly, exendin-4 appears to stimulate cholangiocyte proliferation through the GLP-1 receptor, hereby preventing cholangiocyte apoptosis in models of bile acid-induced damage and models of ductopenic cholangiopathies (95, 96). Although these data are considered beneficial, it also indicates that GLP-1RA could have direct adverse effects on the biliary tree. What the exact mechanism is behind the gallbladder events requires further study, but probably encompasses a combination of factors.

### Cardiovascular

All GLP-1RAs increase heart rate, and this is not different for semaglutide. In SUSTAIN 6, a placebo-corrected heart rate increase of 2.75 beats per minute (bpm) was observed for semaglutide 0.5 mg, and 3.2 bpm for the 1.0 mg dosage (97). This increase was not associated with adverse cardiac events.

In addition, no increase in cardiovascular outcomes were observed in SUSTAIN 6 and PIONEER 6, which is reassuring given the initial fear of adverse cardiac events with increased resting heart rates. Large epidemiological studies have found that an increase in 5 bpm is associated with an increase of 17% in mortality (98). It is unclear whether this association holds true for drug-induced heart-rate acceleration. The α-blocking agent doxazosin increases heart rate by ~25% (99), and is associated with an increase in heart failure incidence (compared with the diuretic agent chlorthalidone) (100). In contrast, lowering heart rate by approximately 10 bpm using the cardiac funny-channel inhibitor ivabradine did not affect mortality in patients with stable coronary artery disease. At this point, it is clear that the beneficial effects of GLP-1RA on cardiovascular risk factors and physiology outweigh a potential risk of the associated heart rate increase. Liraglutide has been on the market for 10 years, but cardiovascular safety beyond this has not been studied yet.

The increase in heart rate is also of importance in patients with heart failure (HF). While the semaglutide CVOTs did not show an increased incidence of hospitalization for HF compared to placebo (101), in earlier smaller studies with liraglutide in patients with HF with reduced left ventricular ejection fraction, the GLP-1RA was associated with increased incidence of serious cardiac events (rhythm disorders, worsening of HF) (102, 103). Since patients with HF with New York Heart Association class IV were excluded from the CVOTs, it is unclear whether safety risks could occur in semaglutide-treated patients. However, a recent meta-analysis of all current CVOTs, showed that GLP-1RAs as a group were associated with a (non-significant) reduction in HF (104).

Several clinical mechanistic trials provided conflicting evidence while aiming to understand the GLP-1RA-induced heart rate-increase. Some studies found systemic vasodilation (with likely consequent reflex tachycardia), while others failed to show this (105–107). Similarly, discrepant findings are available for activation of the (cardiac) sympathetic nervous system (106, 108–111). Our own group previously hypothesized a direct effect of GLP-1RAs on sino-atrial cells (106), after exclusion of other potential causes. This postulation was later confirmed in a mouse model, where stimulation of GLP-1 receptors on atrial cells induced a chronotropic effect, but only when neuronal input was present (112).

Most novel drugs also undergo testing for their effect on the QT interval, as QT prolongation is a marker for potential ventricular fibrillation. Compared with placebo, subcutaneous semaglutide had no effect on this ECG measure in healthy volunteers, with doses above what is used in daily practice (113).

### Acute Kidney Injury

Initial case reports suggested that GLP-1RA treatment could cause acute kidney injury (AKI) in some patients (114). Mechanistically, this was explained by dehydration caused by nausea, vomiting and diarrhea (see above). Also, very recently it was shown that the GLP-1RA, dulaglutide decreased fluid intake (115). Furthermore, GLP-1RA potentially further compromise fluid homeostasis by increasing renal sodium excretion (116). Combined, this could induce renal failure, especially in frail patients or those with medication such as renin–angiotensin–aldosterone system inhibitors, non-steroidal anti-inflammatory drugs or diuretic drugs.

In the SUSTAIN program, acute kidney failure was only reported in SUSTAIN 6, where its occurrence was similar between semaglutide and placebo (28). In PIONEER, AKI was a safety event of interest, and reported in all papers (14, 17–19, 21, 23). In PIONEER 6, AKI occurred in 2.0% of patients treated with oral semaglutide and 2.3% of placebo-treated patients (19). Whether this is statistically or clinically significant has not been evaluated yet.

In contrast to the incidental cases of AKI, the CVOTs mainly demonstrate a beneficial effect on renal outcomes, likely because of effects on cardiovascular risk factors (117). As recently reviewed, GLP-1RAs reduce progression to macro-albuminuria and lead to (subtle) reductions in the decline in renal function (118). In a recent post-hoc analysis of SUSTAIN 6, semaglutide was associated with less events of nephropathy, independent of baseline blood pressure (119). Thus, while it is conceivable yet not statistically confirmed that semaglutide could cause AKI in selected patients, there is plenty of evidence that it reduces nephropathy in the long term. A dedicated kidney trial (the FLOW study; NCT03819153) is currently ongoing, studying the effects of subcutaneous semaglutide on renal outcomes in people with T2D and chronic kidney disease.

### Diabetic Retinopathy

In the SUSTAIN-6 trial, an increase in DRP complications, defined as a composite of need for retinal photocoagulation or treatment with intravitreal agents or vitreous hemorrhage or diabetes-related-blindness, was reported for semaglutide compared to placebo (hazard ratio 1.76; 95% CI 1.11–2.78). In a large systemic review and network analysis, including several GLP-1RAs, subcutaneous semaglutide was the only glucose-lowering drug for which this signal was observed (120). However, in the LEADER trial, a non-significant trend towards DRP was observed for liraglutide (121). In PIONEER 6, unadjudicated DRP occurred in 5.8% of oral semaglutide-treated patients and in 4.8% of the placebo-treated patients (19).

Villsbol and colleagues further investigated the DRP signal in the SUSTAIN program (122). In SUSTAIN-6, nearly 30% of patients had previous documented DRP, with 6% proliferative DRP. This percentage was not surprising given the inclusion of patients with previous cardiovascular disease, usually associated with long-standing diabetes. In semaglutide-treated patients, 3% (*versus* 1.8% in the placebo group) of patients reached an adjudicated endpoint of DRP. Across all DRP categories as indicated above, more events with semaglutide were noted. Participants that were prone to develop DRP had pre-existing DRP, longer diabetes duration, higher HbA_1c_ levels at baseline, and more often used insulin therapy. Particularly, participants with pre-existing DRP who were using insulin therapy had the highest risk for a new DRP event.

This analysis further assessed whether the increase in DRP was a GLP-1 specific effect, or rather caused by a robust and early glucose lowering as suggested by several other studies, where acute and large reductions in glucose concentrations may initially and transiently worsen DRP, yet prevent or delay onset or progression of DRP in the long term (123–128). Patients that met a DRP endpoint had strongest glucose lowering during the trial, independent of their randomization to semaglutide or placebo. A *post-hoc* mediation analysis adjusting for HbA_1c_ reduction at week 16 showed that glucose reduction at this time point explained the increased incidence. Limitations of DRP assessment during the trial were the absence of assessment of retinal changes over time, while the severity was not graded on baseline. Nevertheless, based on the data brought forward, it seems safe to conclude that the phenomenon of early worsening of pre-existing DRP was secondary to the initial and rapid improvement in glycemic control that occurred in SUSTAIN-6. This was confirmed in the recent AngioSafe study which showed no effect of GLP-1RA therapy on angiogenesis and no association between GLP-1 exposure and severe DRP was shown (129).

Currently, a large trial is ongoing assessing the long-term effects of semaglutide on DRP in patients with T2D as primary outcome (FOCUS trial, NCT03811561). This study will provide important data with respect to semaglutide safety on the retina. Until that time, caution should be exercised when using semaglutide in patients with DRP. It may be sensible to perform a fundoscopy prior to semaglutide therapy, and existing DRP should be treated where necessary. In addition, given the strong effects of semaglutide on glucose levels, down titrating insulin will prevent rapid decreases in glucose concentrations thereby reducing the risk of acute DRP worsening.

### Injection-Site and Allergic Reactions

Although every subcutaneous injection can induce injection-site reactions, there are no signals that this is higher with semaglutide compared with placebo (130). In phase 3 studies, any site reaction was present in 0.6% of patients on the 0.5 mg dose, 0.3% on the 1 mg dose, and 0.8% in the comparator groups. The local site reaction includes bruising, discoloration, induration, and pain (130). In SUSTAIN-6, none of these injection site-reactions was considered severe, and it was never a reason to withhold therapy.

Given the immunogenic potential of protein-based drugs, it is important to monitor allergic reactions with GLP-1RAs. Allergic reactions were reported in four patients in the SUSTAIN program. However, at closer inspection, these reactions were more likely caused by the (concomitant) use of angiotensin-converting enzyme inhibitors or an infection (130). Across the phase 3a PIONEER trials, less subjects with oral semaglutide (2.9%) had allergic reactions compared with the comparators (4.6%) (131). No cases of anaphylactic reactions have yet been attributed to semaglutide; one patient using semaglutide had an anaphylactic shock attributed to cefazolin in SUSTAIN-6.

## Effects of Semaglutide Compared to Other GLP-1 Receptor Agonists

The group of GLP-1RA contains several agents, and their adverse effect profile is not identical. This could be due to differences in pharmacokinetic profile (short- *vs* long-acting) and due to structural differences. Exendin-derived agents, i.e. exenatide and lixisenatide, are based on a protein derived from saliva of the Gila monster, and only share roughly 50% of the homology of GLP-1, which could trigger immunogenicity. The more frequent injection site reactions with exenatide once weekly (22%) compared with semaglutide (1.2%) in SUSTAIN-3 could be a consequence of this (22).

The head-to-head studies within the SUSTAIN and PIONEER programs allow some comparison of the adverse effect profile (**Table 2**). With these data, the safety profiles of rare potential events (e.g. pancreatitis, thyroid cancer, kidney injury, etc.) and hypoglycemia risk are comparable for semaglutide, dulaglutide, exenatide once weekly and liraglutide. However, semaglutide appears to be associated with more frequent nausea and vomiting. In SUSTAIN-3, 41.8% of patients with subcutaneous semaglutide had GI adverse effects, compared with 33.3% in exenatide once weekly (22). In SUSTAIN-7, nausea or vomiting occurred similarly for semaglutide 0.5 mg and 1 mg and dulaglutide 1.5 mg (43–48%), yet less frequent with dulaglutide 0.75 mg (33%) (29). In SUSTAIN-10, 21.8% of semaglutide-patients had nausea, compared to 15.7% of liraglutide-patients (32).

For oral semaglutide, the data are similar. In PIONEER-9, oral semaglutide induced nausea in up to 10% of patients, whereas none of the liraglutide patients had nausea (liraglutide was low-dose however) (23). Compared with dulaglutide in PIONEER-10, nausea rates were similar, yet oral semaglutide was more frequently associated with vomiting (14 mg dose semaglutide: 7%, dulaglutide 0.75 mg 2%) (24).

Finally, in a network meta-analysis, several short- and long-acting GLP-1RA were compared regarding efficacy and side effect profile. Compared with lixisenatide, exenatide twice daily, liraglutide, albiglutide and dulaglutide, semaglutide is associated with highest nausea and vomiting rates, yet also with highest rates of improvement in glycemic control and weight loss (132).

Whether the more rare adverse events differ between the different agents can only be answered by using observational cohort data from a very large group of patients and a longer follow-up time. Since semaglutide is relatively new, these data are not available yet. It should be stressed that guidelines do not favor the prescription of one GLP-1 RA over another, although clinicians are advised to select a compound with proven cardiovascular benefit.

## Discussion

Since the finding that the thiazolidinedione rosiglitazone increased cardiovascular events, much weight has been placed on the safety of novel glucose-lowering drugs. For all new drugs, a thorough safety profile needs to be established, with particular emphasis on cardiovascular safety. While safety within the phase 3 program is sufficient for marketing authorization (although the risk of cardiovascular events should not exceed a hazard ratio of 1.8 according to a guidance document that was released by the FDA at that time), it is the post-marketing phase in which rare adverse events and any other potential safety risks are identified or resolved. The same FDA document mandates post-marketing trials to demonstrate that the novel agent does not increase cardiovascular risk by more than 30% compared to placebo (henceforth these trials were named ‘cardiovascular outcome trial’), if premarketing studies did not already demonstrate this.

Although designed for cardiovascular safety, other safety aspects may also be assessed in CVOTs. Moreover, after marketing approval, several databases can be employed to understand safety risks. In this regard, case reports and studies using adverse event databases (such as the FDA Adverse Event Reporting System and European Eudravigilance) frequently are the first signals of potential safety risks. With GLP-1RA, these encompassed AKI, pancreatitis, pancreatic cancer and thyroid cancer (6, 114). While awaiting the CVOTs, results from several health care database studies (insurance claims, hospital registry, etc.) were published, and were somewhat conflicting. With the totality of the evidence, many of the feared safety risks were nuanced or refuted.

As semaglutide is one of the youngest GLP-1RA, it was possible to prospectively monitor for the rarer adverse events in the phase 3 program and CVOT. As detailed in the current review, semaglutide appears not to increase the risk of pancreatitis (yet nevertheless it has been added to the SmPC to align with health authorities expectations on class labeling), but it is associated with more events of cholelithiasis. Although current data argue against an increased risk of pancreatic cancer and thyroid cancer with semaglutide, it can be debated whether the background incidence of these disorders is too low to fully conclude the absence of an association.

Even though the route of administration, their drug formulation and the dosage differ, the AE profile appears not to be very different between subcutaneous and oral semaglutide. One important co-product in oral semaglutide, SNAC, can be toxic at high doses (133). However, at the SNAC dosage of 300 mg per tablet of oral semaglutide, it is well below the toxic dose of 1.8 g/kg/day observed in monkeys, where it caused nausea and diarrhea (133). Post-marketing surveillance will help to elucidate whether the subcutaneous and oral variant differ in their real-world safety profile.

Most data reported in this review are from phase 3 clinical trials. Whether all of these data can be extrapolated to clinical practice remains a matter of debate. In RCTs, there are tightly regulated cohorts based on stringent in- and exclusion criteria, thereby reducing generalizability. Moreover, the frequent visits and calls during a study could improve patient coherence. However, real-world evidence—where available—has not shown major differences in for example hypoglycemia rate or drug discontinuation (36, 39, 40).

## Conclusion

Over the years, the use of GLP-1RAs has first been associated with several adverse events, which were later mostly nuanced or refuted. As one of the newer agents within the class, the safety of semaglutide—both the subcutaneous and oral formulation—has been scrutinized in the phase 3 programs and CVOTs. Compared with placebo and active comparator, semaglutide induces mostly mild and transient gastrointestinal disturbances, and increases the risk of cholelithiasis. However, no major safety concerns have arisen to date, although definitive conclusions for pancreatic cancer, thyroid cancer and DRP complications cannot be drawn at this point. When compared with the beneficial effects of these drugs on glucose metabolism, blood pressure, body weight and cardiovascular (and potentially even renal) endpoints, these agents have an overall beneficial risk/benefit-profile for treatment of patients with T2D.

## Author Contributions

The authors drafted all versions of the article, and provided final approval for submission. All authors contributed to the article and approved the submitted version.

## Funding

This article was supported by Novo Nordisk, who was provided with the opportunity to perform a medical accuracy review.

## Conflict of Interest

DVR has acted as a consultant and received honoraria from Boehringer Ingelheim, Eli Lilly, Merck, Novo Nordisk and Sanofi and has received research operating funds from the Boehringer Ingelheim–Eli Lilly Diabetes Alliance, MSD, AstraZeneca and Novo Nordisk.

The remaining author declares that the research was conducted in the absence of any commercial or financial relationships that could be construed as a potential conflict of interest.

The authors declare that this article received funding from Novo Nordisk. The funder had the following involvement in the article: medical writing support.
