# Corrigendum: Safety of Semaglutide

**DOI:** 10.3389/fendo.2021.786732

**Published:** 2021-11-10

**Authors:** Mark M. Smits, Daniël H. Van Raalte

**Affiliations:** Diabetes Center, Department of Internal Medicine, Amsterdam University Medical Center, Amsterdam, Netherlands

**Keywords:** glucagon-like peptide-1 receptor agonist (GLP-1RA), oral, subcutaneous, semaglutide, type 2 diabetes, safety

In the original article, there was a mistake in **Table 2** ***. Adverse effects and safety risks in phase 3 trials*** as published. **Three values in the row for SUSTAIN 6, s.c semaglutide 1 mg were accidentally transposed to the incorrect column.** The corrected **Table 2** appears below.

**Table 2:** Adverse effects and safety risks in phase 3 trials (10–32).

Treatment arms	Incidence of AE, n (%)											% of patients with AE leading to trial product discontinuation	
	Any	Severe or confirmed symptomatic hypoglycemic episode*	Gastrointestinal			Pancreas		Gallbladder	Thyroid	Diabetic retinopathy	Acute kidney Injury	Any AE, n(%)	Gastrointestinal, %
			Nausea	Vomiting	Diarrhea	Pancreatitis	Pancreatic cancer						
**PIONEER 1**													
Oral semaglutide 3 mg	101 (57.7)	5 (2.9)	14 (8.0)	5 (2.9)	15 (8.6)	0		NR	0	1 (0.6%)	0	4 (2.3)	75
Oral semaglutide 7 mg	93 (53.1)	2 (1.1)	9 (5.1)	8 (4.6)	9 (5.1)	0		NR	0	6 (3.4%)	0	7 (4.0)	57
Oral semaglutide 14 mg	99 (56.6)	1 (0.6)	28 (16.0)	12 (6.9)	9 (5.1)	0		NR	0	2 (1.1%)	1 (0.6)	13 (7.4)	69
Placebo	99 (55.6)	1 (0.6)	10 (5.6)	4 (2.2)	4 (2.2)	0		NR	0	3 (1.7%)	1 (0.6)	4 (2.2)	25
**PIONEER 2**													
Oral semaglutide 14 mg	289 (70.5)	7 (1.7)	81 (19.8)	30 (7.3)	38 (9.3)	1 (0.2)	0	NR	0	14 (3.4)	2 (0.5)	44 (10.7)	75
Empagliflozin 25 mg	283 (69.2)	8 (2.0)	10 (2.4)	7 (1.7)	13 (3.2)	1 (0.2)	0	NR	0	5 (1.2%)	1 (0.2)	18 (4.4)	17
**PIONEER 3**													
Oral semaglutide 3 mg	370 (79.4)	23 (4.9)	34 (7.3)	13 (2.8)	45 (9.7)	1 (0.2)	0	NR	0	27 (5.8)	3 (0.6)	26 (5.6)	42
Oral semaglutide 7 mg	363 (78.2)	24 (5.2)	62 (13.4)	28 (6.0)	53 (11.4)	1 (0.2)	0	NR	0	24 (5.2)	2 (0.4)	27 (5.8)	56
Oral semaglutide 14 mg	370 (79.6)	36 (7.7)	70 (15.1)	42 (9.0)	57 (12.3)	1 (0.2)	1 (0.2)	NR	0	16 (3.4)	5 (1.1)	54 (11.6)	59
Sitagliptin 100 mg	388 (83.3)	39 (8.4)	32 (6.9)	19 (4.1)	37 (7.9)	1 (0.2)	1 (0.2)	NR	0	27 (5.8)	3 (0.6)	24 (5.2)	50
**PIONEER 4**													
Oral semaglutide 14 mg	229 (80)	2 (1)	56 (20)	25 (9)	43 (15)	0	0	NR	1 (0.4)	8 (3)	0	31 (11)	71
Liraglutide 1.8 mg (s.c.)	211 (74)	7 (2)	51 (18)	13 (5)	31 (11)	1 (0.4)	1 (0.4)	NR	1 (0.4)	4 (1)	1 (0.4)	26 (9)	65
Placebo	95 (67)	3 (2)	5 (4)	3 (2)	11 (8)	1 (0.7)	0	NR	0	2 (1)	1	5 (4)	60
**PIONEER 5**													
Oral semaglutide 14 mg	122 (75)	9 (6)	31 (19)	19 (12)	17 (10)	0	0	NR	0	5 (3)	3 (1.8)	24 (15)	79
Placebo	109 (68)	3 (2)	12 (7)	6 (4)	6 (4)	0	0	NR	0	2 (1)	1 (0.6)	8 (5)	38
**PIONEER 6**													
Oral semaglutide 14 mg	NR	NR	NR	NR	NR	1 (0.1)	0	NR	2 (0.1)	93 (5.8)	32 (2.0)	184 (11.6)	59
Placebo	NR	NR	NR	NR	NR	3 (0.2)	0	NR	0	76 (4.8)	37 (2.3)	104 (6.5)	25
**PIONEER 7**													
Oral semaglutide (flexible 3, 7 or 14 mg)	197 (78)	14 (5.5)	53 (21)	14 (6)	22 (9)	0	0	NR	0	6 (2.4)	1 (0.4)	22 (9)	64
Sitagliptin 100 mg	172 (69)	14 (5.6)	6 (2)	3 (1)	8 (3)	0	0	NR	0	6 (2.4)	0	8 (3)	25
**PIONEER 8**													
Oral semaglutide 3 mg	137 (74.5)	52 (28.3)	21 (11.4)	11 (6.0)	16 (8.7)	0	0	NR	0	7 (3.8)	2 (1.1)	13 (7.1)	69
Oral semaglutide 7 mg	142 (78.5)	47 (26.0)	30 (16.6)	14 (7.7)	22 (12.2)	0	0	NR	0	8 (4.4)	1 (0.6)	16 (8.8)	75
Oral semaglutide 14 mg	151 (83.4)	48 (26.5)	42 (23.2)	18 (9.9)	27 (14.9)	0	0	NR	0	9 (5.0)	0	24 (13.3)	79
Placebo	139 (75.5)	54 (29.3)	13 (7.1)	7 (3.8)	11 (6.0)	0	0	NR	0	8 (4.3)	0	5 (2.7)	20
**PIONEER 9**													
Oral semaglutide 3 mg	37 (76)	0	2 (4)	NR	4 (8)	0	0	NR	0	0	0	1 (2)	100
Oral semaglutide 7 mg	37 (76)	0	5 (10)	NR	1 (2)	0	0	NR	1	1 (2.0)	0	1 (2)	100
Oral semaglutide 14 mg	34 (71)	0	4 (8)	NR	3 (6)	0	0	NR	0	1 (2.1)	0	2 (4)	100
Liraglutide 0.9 mg (s.c.)	32 (67)	2 (4.2)	0	NR	2 (4)	0	0	NR	0	0	0	0	0
Placebo	39 (80)	0	1 (2)	NR	1 (2)	0	0	NR	0	2 (4.1)	0	0	0
**PIONEER 10**													
Oral semaglutide 3 mg	101 (77)	3 (2)	7 (5)	3 (2)	2 (2)	0	0	2 (2)	0	9 (7)	0	4 (3)	50
Oral semaglutide 7 mg	106 (80)	3 (2)	11 (8)	6 (5)	2 (2)	0	0	1 (1)	0	12 (9)	0	8 (6)	50
Oral semaglutide 14 mg	111 (85)	4 (3)	12 (9)	9 (7)	10 (8)	0	0	0	0	7 (5)	0	8 (6)	63
Dulaglutide 0.75 mg (s.c.)	53 (82)	0	6 (9)	1 (2)	4 (6)	0	0	1 (2)	0	3 (5)	0	2 (3)	50
**SUSTAIN 1**													
S.c. semaglutide 0.5 mg	82 (64)	0	26 (20)	5 (4)	16 (13)	0	0	3 (2)	0	NR	0	8 (6)	63
S.c. semaglutide 1 mg	73 (56)	0	31 (24)	9 (7)	14 (11)	0	0	1 (<1)	0	NR	0	7 (5)	57
Placebo	69 (53)	3 (2)	10 (8)	2 (2)	3 (2)	0	0	0	0	NR	0	3 (2)	33
**SUSTAIN 2**													
S.c. semaglutide 0.5 mg	306 (75)	7 (2)	73 (18)	33 (8)	54 (13)	3 (1%)	NR	1 (<1)	0	1 (<1)	NR	33 (8)	82
S.c. semaglutide 1 mg	292 (71)	2 (<1)	72 (18)	41 (10)	53 (13)	1 (<1)	NR	7 (2)	1	0	NR	39 (10)	79
Sitagliptin 100	292 (72)	5 (1)	30 (7)	11 (3)	29 (7)	0	NR	6 (1)	0	3 (1)	NR	12 (3)	25
**SUSTAIN 3**													
S.c. semaglutide 1 mg	303 (75)	33 (8.2)	90 (22.3)	29 (7.2)	46 (11.4)	2 (<1)	NR	6 (1%)	NR	NR	NR	38 (9.4)	NR
Exenatide ER 2.0 mg	309 (76.3)	33 (8.1)	48 (11.9)	25 (6.2)	34 (8.4)	3 (<1)	NR	2 (<1)	NR	NR	NR	29 (7.2)	NR
**SUSTAIN 4**													
S.c. semaglutide 0.5 mg	253 (70)	16 (4)	77 (21)	24 (7)	59 (16)	2 (1)	1 (<1)	1 (<1)	NR	1 (<1)	NR	20 (6)	55
S.c. semaglutide 1 mg	264 (73)	20 (6)	80 (22)	37 (10)	69 (19)	0	0	2 (1)	NR	0	NR	27 (8)	70
Insulin glargine	235 (65)	38 (11)	13 (4)	11 (3)	16 (4)	0	0	0	NR	1 (<1)	NR	4 (1)	0
**SUSTAIN 5**													
S.c. semaglutide 0.5 mg	91 (68.9)	11 (8.3)	15 (11.4)	8 (6.1)	6 (4.5)	0	0	3 (2.3)	0	(3.0)	NR	6 (4.5)	NR
S.c. semaglutide 1 mg	84 (64.1)	14 (10.7)	22 (16.8)	15 (11.5)	9 (6.9)	0	0	1 (0.8)	0	(0.8)	NR	8 (6.1)	NR
Placebo	77 (57.9)	7 (5.3)	6 (4.5)	4 (3.0)	2 (1.5)	0	0	0	0	0	NR	1 (0.8)	NR
**SUSTAIN 6**													
S.c. semaglutide 0.5 mg	740 (89.6)	191 (23.1)	143 (17.3)	14 (1.7)	15 (1.8)	6 (0.7)	0	25 (3)	0		42 (5.1)	95 (11.5)	49
S.c. semaglutide 1 mg	732 (89.1)	178 (21.7)	180 (21.9)	23 (2.8)	19 (2.3)	3 (0.4)	1 (0.1)	17 (2.1)	0	50 (3.0)	23 (2.8)	119 (14.5)	65
Placebo	1484 (90)	350 (21.2)	129 (7.8)	5 (0.3)	7 (0.4)	12 (0.7)	4 (0.2)	39 (2.3)	0	29 (1.8)	34 (4.1)	110 (6.7)	16
**SUSTAIN 7**													
S.c. semaglutide 0.5 mg	204 (68)	2 (1)	68 (23)	31 (10)	43 (14)	0	0	2 (1)	1 (<1)	2 (1)	NR	24 (8)	67
S.c. semaglutide 1 mg	207 (69)	5 (2)	63 (21)	31 (10)	41 (14)	0	0	4 (1)	0	2 (1)	NR	29 (10)	62
Dulaglutide 0.75 mg (s.c.)	186 (62)	3 (1)	39 (13)	12 (4)	23 (8)	0	0	4 (1)	0	2 (1)	NR	14 (5)	43
Dulaglutide 1.5 mg (s.c.)	221 (74)	5 (2)	60 (20)	29 (10)	53 (18)	0	0	8 (3)	1 (<1)	3 (1)	NR	20 (7)	70
**SUSTAIN 8**													
S.c. semaglutide 1 mg	298 (76)	53 (14)	89 (23)	50 (13)	60 (15)	NR	NR	NR	NR	9 (2)	4 (1)	38 (10)	68
Canagliflozin 300 mg	283 (72)	32 (8)	26 (7)	9 (2)	37 (9)	NR	NR	NR	NR	15 (4)	0	20 (5)	20
**SUSTAIN 9**													
S.c. semaglutide 1 mg	104 (69.3)	17 (11.3)	29 (19.3)	14 (9.3)	17 (11.3)	0	0	NR	NR	3 (2.0)	1 (0.7)	13 (8.7)	77
Placebo	91 (60.3)	3 (2.0)	5 (3.3)	3 (2.0)	9 (6.0)	0	0	NR	NR	8 (5.3)	0	3 (2.0)	0
**SUSTAIN 10**													
S.c. semaglutide 1 mg	204 (70.6)	5 (1.7)	63 (21.8)	30 (10.4)	45 (15.6)	0	NR	NR	NR	3 (1.0)	NR	33 (11.4)	67
Liraglutide 1.2 mg (s.c.)	190 (66.2)	7 (2.4)	45 (15.7)	23 (8.0)	35 (12.2)	2 (0.7%)	NR	NR	NR	4 (1.4)	NR	19 (6.6)	58
**SUSTAIN JAPAN 'SITA'**													
S.c. semaglutide 0.5 mg	77 (74.8)	0	(10.7)		(6.8%)	0	0	1 (1.0)	0	4 (3.9)	NR	3 (2.9)	NR
S.c. semaglutide 1 mg	73 (71.6)	1 (1.0)	(12.7)		(8.8%)	0	0	3 (2.9)	0	2 (1.9)	NR	11 (10.8)	NR
Sitagliptin 100 mg	68 (66.0)	0	0		(1.9%)	0	1 (1.0)	0	0	4 (3.9)	NR	2 (1.9)	NR
**SUSTAIN JAPAN 'INDIVIDUAL'**													
S.c. semaglutide 0.5 mg	206 (86.2)	3 (1.3)	29 (12.1)	13 (5.4)	24 (10.0)	0	0	4 (1.7%)	0	11 (4.6)	NR	14 (5.9)	NR
S.c. semaglutide 1 mg	212 (88)	6 (2.5)	46 (19.1)	14 (5.8)	38 (15.8)	0	0	2 (0.8%)	0	16 (6.6)	NR	26 (10.8)	NR
Additional OAD (investigators discretion)	86 (71.7)	2 (1.7)	1 (0.8)	2 (1.7)	8 (6.7)	0	0	0	0	6 (5.0)	NR	4 (3.3)	NR
**SUSTAIN China**													
S.c. semaglutide 0.5 mg	209 (72.8%)	2 (0.7%)	22 (7.7%)	14 (4.9%)	58 (20.2%)	0	0	NR	NR	19 (6.6%)	NR	17 (5.9%)	59
S.c. semaglutide 1 mg	216 (74,5%)	6 (2.1%)	39 (13.4%)	19 (6.6%)	49 (16.9%)	1 (0.3%)	0	NR	NR	14 (4.8%)	NR	31 (10.7%)	68
Sitagliptin 100 mg	199 (68,6%)	4 (1.4%)	5 (1.7%)	3 (1.0%)	20 (6.9%)	0	0	NR	NR	10 (3.4%)	NR	6 (2.1%)	17

AE, adverse event; ER, extended release; NR, not reported; OAD, oral antidiabetic drug; s.c. subcutaneous.

An independent external adjudication committee (EAC) validated prespecified categories of adverse events (including deaths, selected cardiovascular events, malignant neoplasms, thyroid diseases [malignant thyroid neoplasms and C-cell hyperplasia], acute kidney injury, acute pancreatitis, and lactic acidosis) except in SUSTAIN 10 where there was no adjudication.

*An episode that was severe according to the ADA classification (requires assistance of another person to actively administer carbohydrate, glucagon, or other corrective action) or an episode with confirmed blood glucose value <56 mg/dL and symptoms consistent with hypoglycemia.

The authors apologize for this error and state that this does not change the scientific conclusions of the article in any way. The original article has been updated.

## Publisher’s Note

All claims expressed in this article are solely those of the authors and do not necessarily represent those of their affiliated organizations, or those of the publisher, the editors and the reviewers. Any product that may be evaluated in this article, or claim that may be made by its manufacturer, is not guaranteed or endorsed by the publisher.

